# Release of endothelial cell associated VEGFR2 during TGF-β modulated angiogenesis *in vitro*

**DOI:** 10.1186/s12860-017-0127-y

**Published:** 2017-01-23

**Authors:** M. Jarad, E. A. Kuczynski, J. Morrison, A. M. Viloria-Petit, B. L. Coomber

**Affiliations:** Department of Biomedical Sciences, Ontario Veterinary College, University of Guelph, OVC Room 3645, Guelph, N1G 2W1 ON Canada

**Keywords:** Tip cell, Vascular sprouting, Notch1, Dll4, Smad signaling, Microparticle

## Abstract

**Background:**

Sprouting angiogenesis requires vascular endothelial proliferation, migration and morphogenesis. The process is regulated by soluble factors, principally vascular endothelial growth factor (VEGF), and via bidirectional signaling through the Jagged/Notch system, leading to assignment of tip cell and stalk cell identity. The cytokine transforming growth factor beta (TGF-β) can either stimulate or inhibit angiogenesis via its differential surface receptor signaling. Here we evaluate changes in expression of angiogenic signaling receptors when bovine aortic endothelial cells were exposed to TGF-β1 under low serum conditions.

**Results:**

TGF-β1 induced a dose dependent inhibition of tip cell assignment and subsequent angiogenesis on Matrigel, maximal at 5.0 ng/ml. This occurred via ALK5-dependent pathways and was accompanied by significant upregulation of the TGF-β co-receptor endoglin, and SMAD2 phosphorylation, but no alteration in Smad1/5 activation. TGF-β1 also induced ALK5-dependent downregulation of Notch1 but not of its ligand delta-like ligand 4. Cell associated VEGFR2 (but not VEGFR1) was significantly downregulated and accompanied by reciprocal upregulation of VEGFR2 in conditioned medium. Quantitative polymerase chain reaction analysis revealed that this soluble VEGFR2 was not generated by a selective shift in mRNA isoform transcription. This VEGFR2 in conditioned medium was full-length protein and was associated with increased soluble HSP-90, consistent with a possible shedding of microvesicles/exosomes.

**Conclusions:**

Taken together, our results suggest that endothelial cells exposed to TGF-β1 lose both tip and stalk cell identity, possibly mediated by loss of VEGFR2 signaling. The role of these events in physiological and pathological angiogenesis requires further investigation.

**Electronic supplementary material:**

The online version of this article (doi:10.1186/s12860-017-0127-y) contains supplementary material, which is available to authorized users.

## Background

The cytokine transforming growth factor beta (TGF-β) is a member of the TGF-β superfamily consisting of 33 members including TGF-βs (1–3) [1]. TGF-β1 is the predominant and more ubiquitous form. TGF-β ligands signal canonically through type I (ALK) and type II serine/threonine kinase receptors [2], and via the accessory (type III) receptor endoglin in vascular endothelial cells [3]. TGF-β binding leads to phosphorylation of intracellular R-Smads 1, 2, 3, 5 or 8 [2, 4], which then complex with Co-Smad4, enter the nucleus and associate with transcriptional co-activators or co-repressors to regulate the expression of target genes.

TGF-β plays a dual role in angiogenesis by orchestrating a switch from vascular inhibition to pro-angiogenic activity [5–7]. In particular, there is evidence that TGF-β1 induced angiogenesis acts with VEGF to mediate apoptosis of excessive vascular sprouts and may even be required for initial sprouting from an existing vascular network [8, 9]. The nature of the angiogenic response to TGF-β depends on the balance of ALK1 versus ALK5 signaling input, with ALK1 predominantly promoting sprouting and ALK5 favoring the resolution/stabilization phase of angiogenesis [6]. Thus, TGF-β is either pro- or anti-angiogenic, depending on which TGF-RII/Smad pathway is engaged [10]. Differences also exist in the kinetics and dose responses of these pathways: ALK1 mediated signaling is transient and maximal at low concentrations of ligand while ALK5 mediated signaling is sustained and maximal at higher doses of ligand [11]. Increased expression of endoglin led to inhibition of TGF-β/ALK5 signaling (as demonstrated by Smad and CAGA dependent reporter activity) in a dose dependent fashion [12].

Activation of vascular sprouting during angiogenesis entails the specification of endothelial cells into tip and stalk cells. Endothelial tip cells are mainly migratory and polarized with minimal proliferation while stalk cells proliferate throughout sprout establishment and form the nascent vascular lumen cells [13]. The delta-like ligand 4 (Dll4)-Notch1 signaling pathway is involved in tip-stalk cell identity [14]. Tip cells express high levels of Dll4 and vascular endothelial growth factor receptor-2 (VEGFR2), and have low levels of Notch signaling activity [13, 15, 16]. The specification of endothelial cells as tip or stalk cells is transient and its reversibility is contingent on the balance between pro-angiogenic factors. Here we quantify the effect of TGF-β1 on in vitro angiogenesis using a Matrigel cord formation assay, and determine the impact of this angiogenic cytokine on expression of molecules associated with endothelial tip and stalk cell identity.

## Methods

### Cells and culture conditions

Primary bovine aortic endothelial cells (BAEC) were isolated and characterized as described [17] and used between passage 4 and 10. Cultures were maintained in Dulbecco’s Modified Eagle Medium (DMEM; Sigma-Aldrich) with 10% fetal bovine serum (FBS; Invitrogen) and 1 mM sodium pyruvate (Sigma-Aldrich) at 37 °C in 5% CO_2_ and 95% atmospheric air. Confluent monolayers were serum starved 16 h prior to treatment.

### TGF-β1 dose-response

Serum starved BAEC were treated with 0 (Control), 0.1, 1.0, 5.0, or 10.0 ng/ml of recombinant human (rh) TGF-β1 (R&D Systems) in serum free DMEM for up to 24 h, ± 5 μM SB-431542 (an inhibitor of TGβR-I isoforms ALK-5, −4 and −7 [18]). Cell supernatant (conditioned medium) was collected and centrifuged at 350 × *g* for 4 min, and adherent cells lysed for protein or RNA analysis. For some assays, conditioned medium was subjected to additional ultracentrifugation (100,000 × *g* for 1 h at 4 °C) prior to western blotting.

### Protein isolation and western blotting

Cells were lysed on ice with cell lysis buffer (Cell Signaling) supplemented with 1% phosphatase inhibitor cocktail 2 (Sigma-Aldrich), 2 μg/ml aprotinin (Sigma-Aldrich) and 1 mM PMSF (Sigma-Aldrich). Total protein was quantified using the Bio-Rad system and electrophoresis was performed with 20–30 μg protein plus β-mercaptoethanol; samples were heated prior to SDS-PAGE electrophoresis. Proteins were transferred to PVDF membrane, blocked in 5% skim milk or 5% BSA for 1 h at room temperature, then incubated in primary antibody overnight at 4 °C. The antibodies used were: mouse monoclonal anti-Adam 10 (sc-48400; 1:200) and goat polyclonal anti-Adam 17 (sc-6416; 1:200); both Santa Cruz Biotechnology; rabbit monoclonal anti-endoglin (#4335; 1:1000), anti-Notch1 (#4380; 1:1000), anti-VEGFR2 (#9698; 1:1000), anti-pVEGFR2 Tyr 951 (#4991; 1:1000), rabbit polyclonal anti-HSP90 (α and β isoforms; #4874; 1:1000) and anti-pSMAD2 (#3101; 1:500), mouse monoclonal anti-SMAD2 (#3103; 1:1000), all from Cell Signaling; rabbit polyclonal anti-Dll4 (ab7280; 1:1000) and anti-MMP-14 (ab73879; 1:500), rabbit monoclonal anti-VEGFR1 (ab32152; 1:10000), all from abcam; anti-α Tubulin (T5168; 1:200000, Sigma-Aldrich). Membranes were washed and incubated with a secondary antibody (goat anti-mouse HRP or goat anti-rabbit HRP; 1:10,000; both Sigma-Aldrich) for 30 min at room temperature. Bands were visualized with Luminata Classico or Luminata Forte Western HRP Substrate (Millipore) and membranes imaged using a ChemiDoc MP Imaging System (Bio- Rad). Protein loading was normalized by stripping and re-probing for α-tubulin, followed by densitometry analysis.

### RNA isolation and gene expression analysis

Cells were lysed in Ribozol (AMRESCO, VWR International, Mississauga, ON) and RNA isolated using Aurum Total RNA columns (Bio-Rad, Mississauga, ON) according to manufacturers’ protocols. RNA was reverse-transcribed with 4 μL iScript (BioRad, Mississauga, ON) before amplification using primers for target and housekeeping genes (Table 1). Ssofast EvaGreen Supermix (BioRad) was used to determine primer efficiency and for quantitative PCR. No-template controls and quantitative PCR reactions were run in triplicate with an initial 2 min denaturation at 95 °C, 40 cycles of 95 °C for 5 s, 60 °C for 5 s, followed by melt curve analysis of 65 °C to 95 °C in 0.5 °C increments on the CFX96 RealTime System (Bio-Rad). Full length and soluble splice variant mRNA for VEGFR2 were first normalized to both HPRT and GAPDH housekeeping genes, and then results from TGF-β1-treated samples were expressed as relative amounts normalized to control (untreated) samples. Data were analyzed using CFX Manager (Bio-Rad).

**Table 1:** Sequences of primers used for qPCR

Gene		Primer sequence
sVEGFR2^a^	F	GCTTTGCTCAGGACAGGAAGAC
	R	GGTCCAGAGTGACTGCCCTA
VEGFR2^a^	F	GCTTTGCTCAGGACAGGAAGAC
	R	CATGCGCTCTAGGACTGTGA
HPRT^b^	F	CGCGCCAGCCGGCTACGTTA
	R	GGCCACAATGTGATGGCCACCC
GAPDH^b^	F	CAGCAACAGGGTGGTGGACC
	R	AGTGTGGCGGAGATGGGGCA

^a^bovine ortholog of human primer sequences derived from [25]

^b^generated using PrimerBLAST http://www.ncbi.nlm.nih.gov/tools/primer-blast/index.cgi?LINK_LOC=BlastHome

### Matrigel cord formation assay

The impact of TGF-β1 and inhibitors on in vitro angiogenesis was determined by cord formation assay. 10 μl of growth factor containing BD Matrigel Matrix (cat # 354234; BD Biosciences) was added to each well of cold angiogenesis μ-slides (ibidi) and allowed to gel at 37 °C for 30 min. 50 μl of BAEC cell suspension containing 1 × 10^4^ cells in Media-200 made with complete supplements (GIBCO) plus or minus TGF-β1 and inhibitors were added to each well. Slides were incubated for 8 h at 37 °C, and one image/well was captured with phase contrast microscopy using a 4X objective. The generation of cord networks was quantified using the tube formation assay analysis service Wimtube (http://www.wimasis.com; ibidi); analyzed parameters were total cord length, # of branching points, and # of loops. # of tip cells was also determined by manual count of phase contrast images; tip cells were defined as free-ended cells/sprouts with visible filopodia.

### Statistical analysis

All statistical analysis and graphing were performed using GraphPad Prism 6 software (GraphPad Software). Means for at least three biological replicates were calculated and plotted with standard error bars. Data were analyzed by using the nonparametric Kruskal-Wallis test followed by Dunn’s *post hoc* test for multiple comparisons, as appropriate. Differences between means were considered statistically significant when the *p* value was less than 0.05. Assays were performed in triplicate unless otherwise indicated.

## Results

**Fig. 1:**
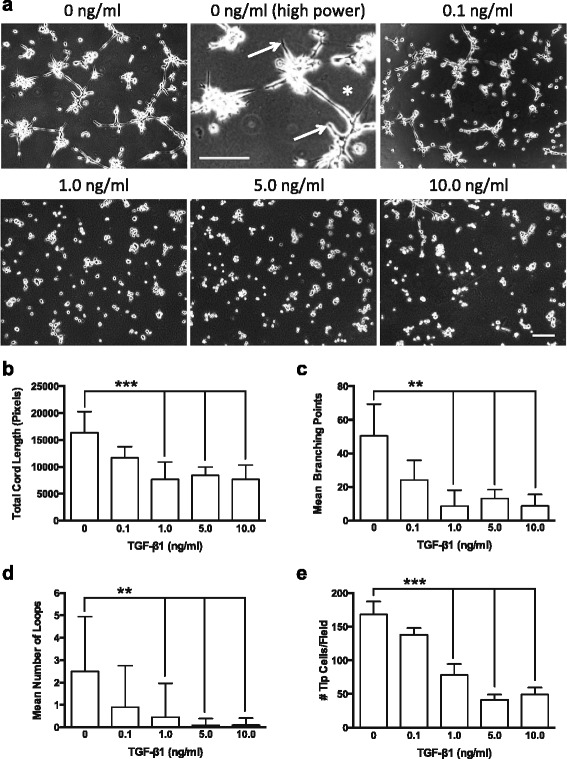
TGF-β1 induced dose-dependent reduction in endothelial cord formation. **a** Representative phase contrast images showing endothelial cell cord formation 8 h after plating on Matrigel™ in the presence of exogenous TGF-β1 (0–10 ng/ml). Note higher magnification control panel (0 ng/ml TGF-β1), showing tip cells (*arrows*) and loop of endothelial cords (*asterisk*). WimTube automated quantification of cord formation showed that TGF-β1 significantly inhibits total cord length (**b**), cord branching (**c**), formation of loops (**d**), and generation of tip cells (**e**). TGF-β1 doses of 1.0 ng/ml or higher significantly inhibited cord formation compared to control (0 ng/ml) or 0.1 ng/ml. **p ≤ 0.01; **p ≤ 0.001; *N* = 4; Kruskal-Wallis test. Scale bars = 300 μm

**Fig. 2:**
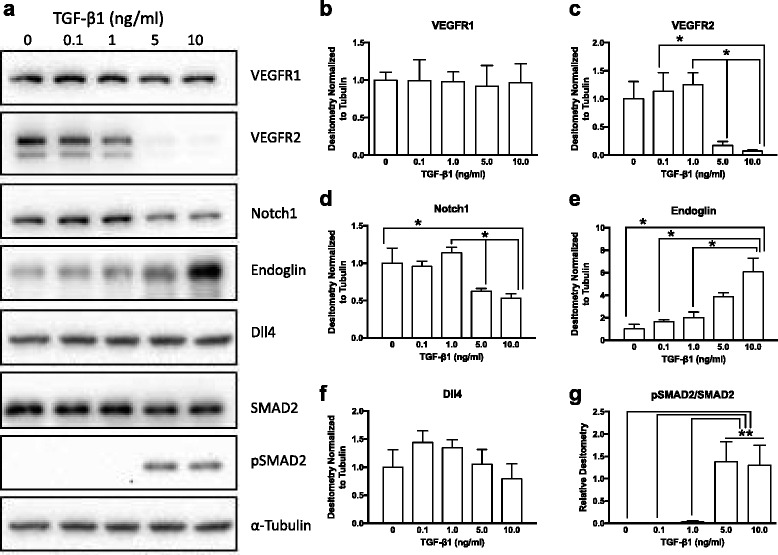
TGF- β1 induced dose-dependent changes in endothelial cell receptors. **a** Representative western blots of endothelial cells exposed to exogenous TGF-β1 (0.1–10.0 ng/ml) for 24 h. Densitometry showed significantly reduced VEGFR2 expression (**b**), but no change in VEGFR1 (**c**) in cells treated with higher doses of TGF-β1. The TGF-β1 co-receptor endoglin was significantly upregulated in a dose dependent fashion (**e**). Notch1 was significantly downregulated in endothelial cells treated with 5.0 and 10.0 ng/ml TGF-β1 (**d**), and its ligand Dll4 showed a trend towards reduced expression with higher doses (**f**). TGF-β1 signaling through ALK5/SMAD2-dependent pathways, as revealed by phosphorylation of SMAD2, was maximal at doses of 5.0 ng/ml and higher (**g**). *p ≤ 0.05; **p ≤ 0.01; *N* = 3; Kruskal-Wallis test

Exogenous TGF-β1 induced a dose-dependent decrease in endothelial cord formation on Matrigel, with significant reductions in cord length, cord branching, loop formation and tip cell generation detectable with doses of TGF-β1 ≥ 1.0 ng/ml (Fig. 1). Western blotting of whole cell lysates from BAEC treated with exogenous TGF-β1 under serum free conditions confirmed our previous findings [19] of significant downregulation of VEGFR2, and no alteration in VEGFR1 expression (Fig. 2 a-c). VEGFR2 was detected as a doublet, with one band approximately 230 kDa (thought to represent the full-length mature transmembrane form), and a smaller (~180–200 kDa) putative ‘immature’ isoform [20]. Reduction in cell associated VEGFR2 protein was seen in both mature and immature forms, and was sustained for at least 24 h after removal of exogenous TGF-β1 (Additional file 1: Figure S1). There was also significant reduction in the levels of Notch1 but no changes in its ligand Dll4 (Fig. 2 a, d, f). TGF-β1 induced a profound upregulation of the type III receptor endoglin (Fig. 2 a, e) and concomitant activation of SMAD2 signaling, as revealed by enhanced levels of phosphorylated SMAD2 (pSMAD2) (Fig. 2 a, g). SMAD2 activation was maximal at 5.0 ng/ml TGF-β1, thus subsequent assays were performed with this concentration.

**Fig. 3:**
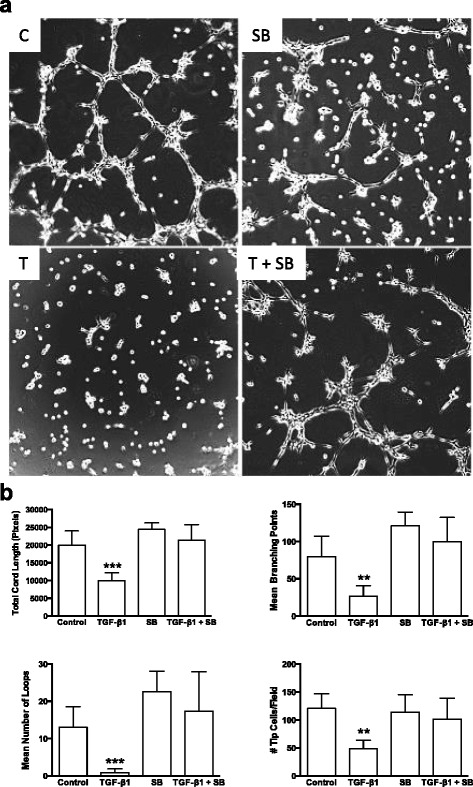
TGF-β1 induced reduction in endothelial cord formation occurs via an ALK5 related pathway. **a** Representative phase contrast images showing endothelial cell cord formation 8 h after plating on Matrigel™ under control conditions (C; DMSO vehicle), 5 μM SB-431542 (an ALK5 inhibitor) in DMSO (SB), 5 ng/ml exogenous TGF-β1 (T) or 5.0 ng/ml TGF-β1 plus 5 μM SB-431542 (T + SB). **b** WimTube automated quantification of cord formation showed SB inhibitor significantly blocked the ability of 5.0 ng/ml TGF-β1 to significantly inhibit total cord length, cord branching, formation of loops, and generation of tip cells. SB inhibitor, either alone or in combination with 5.0 ng/ml TGF-β1 was not significantly different from DMSO control. **p ≤ 0.01; ***p ≤ 0.001; *N* = 4; Kruskal-Wallis test

**Fig. 4:**
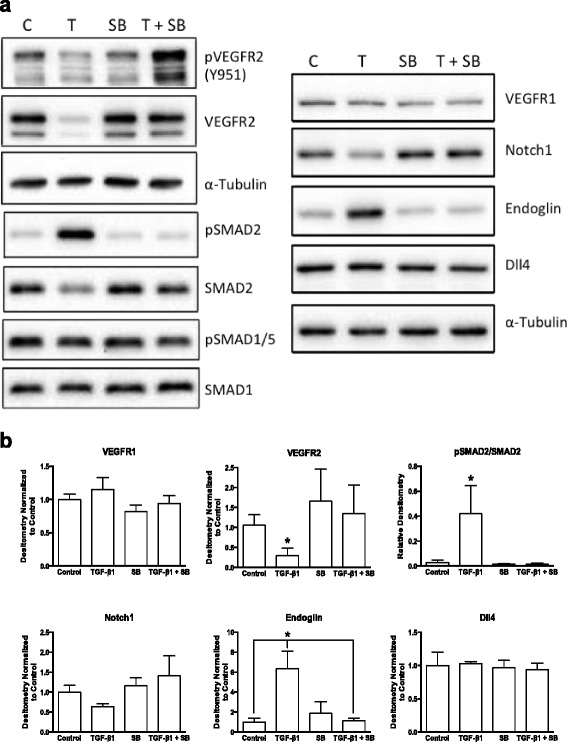
TGF-β1 induced reduction in receptor expression occurs via an ALK5 related pathway. **a**) Representative western blots and densitometry (**b**) from BAEC exposed to control conditions (C; DMSO vehicle), 5 μM SB-431542 (an ALK5 inhibitor) in DMSO (SB), 5 ng/ml exogenous TGF-β1 (T) or 5.0 ng/ml TGF-β1 plus 5 μM SB-431542 (T + SB) for 24 h. TGF-β1 leads reduced VEGFR2 expression and activation, and significantly upregulated endoglin in an ALK5/SMAD2-dependent fashion (as revealed by pSMAD2). There were no significant changes in SMAD1/5-dependent signaling, or in VEGFR1, Notch1 or Dll4 expression. *p ≤ 0.05; *N* = 3; Kruskal-Wallis test

The mechanisms of the observed changes in angiogenic signaling pathways were then investigated. As demonstrated by treatment with the ALK5 signaling inhibitor SB-431542 (SB, 5 μM), blockade of ALK5 signaling significantly eliminated the ability of 5.0 ng/ml TGF-β1 to inhibit Matrigel angiogenesis (Fig. 3 a). SB inhibitor alone had no significant effect compared to DMSO vehicle (Control) in these assays. Exogenous TGF-β1 led to a reduction in VEGFR2 phosphorylation (as revealed by western blotting for pVEGFR2 at residue tyrosine 951) and expression levels, but addition of the SB inhibitor rescued VEGFR2 signaling (Fig. 4 a). Similar results were obtained using SD-208, another ALK5 signaling inhibitor (Additional file 2: Figure S2). TGF-β1 activation of SMAD2 phosphorylation and endoglin upregulation was also blocked by SB (Fig. 4). Notch1 levels showed a trend towards significant differences with SB inhibitor (*p* = 0.07), but expression of Dll4 was not altered (Fig. 4). SMAD1/5 phosphorylation was also not altered in these samples. Thus, ALK5 appeared to mediate the effects of TGF-β1 on the observed altered angiogenesis signaling pathways.

**Fig. 5:**
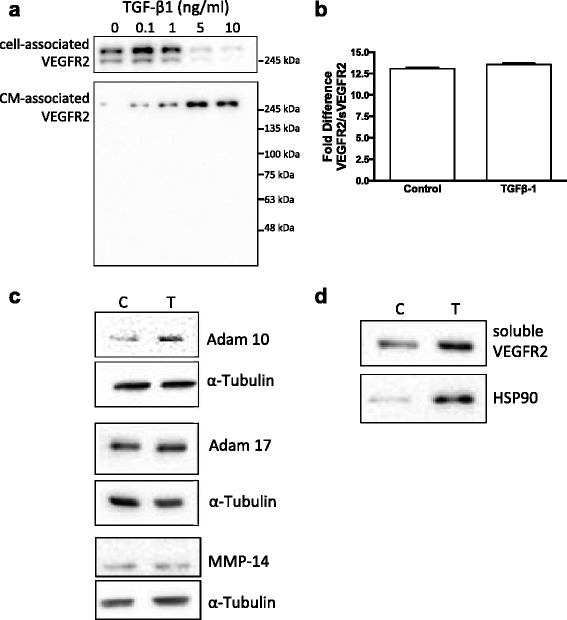
TGF-β1 reduces VEGFR2 expression via multiple mechanisms. **a** Western blot showing TGF-β1 induced loss of cell associated VEGFR2 protein and concomitant increase in full-length VEGFR2 detected in conditioned medium. **b** Quantitative PCR analysis showed that there was no change in the relative ratio of full length VEGFR2 mRNA to alternative spliced sVEGFR2 mRNA upon TGF-β1 treatment. **c** TGF-β1 treatment induced increased expression of ADAM family sheddase enzyme ADAM 10. TGF-β1 treatment had no effect on the expression of ADAM 17 or the membrane-associated metalloproteinase MMP-14. **d** Ultracentrifugation of endothelial conditioned medium demonstrated that soluble VEGFR2 is associated with the extracellular vesicle/exosome marker HSP90 in TGF-β1 treated cells

The basis of VEGFR2 downregulation was further investigated. Loss of VEGFR2 from whole cell lysates of TGF-β1 treated BAEC was accompanied by concomitant increasing levels of full-length protein in serum free conditioned media (CM) collected from the same cells (Fig. 5 a). qPCR analysis showed that both full-length and the soluble VEGFR2 (sVEGFR2) splice variant mRNAs were expressed by these cells, with the full-length isoform being the predominant species, expressed approximately 16 fold higher than the sVEGFR2 message. Both isoforms of VEGFR2 message were significantly downregulated in TGF-β1 treated cells compared to control. However, the ratio of full-length to soluble VEGFR2 mRNA was not altered by 5.0 ng/ml TGF-β1 treatment (Fig. 5 b), supporting the finding from Fig. 5a that a truncated soluble form of VEGFR2 protein is not released from these cells upon TGF- β1 exposure.

No change in MMP-14 (MT1-MMP) or ADAM17 protein levels were detected, and ADAM10 was weakly upregulated in TGF-β1 treated cells, suggesting enzymatic shedding was not the mechanism for increased CM-associated VEGFR2 (Fig. 5 d). In contrast, increased levels of the extracellular vesicle/exosome-associated protein HSP90 were also detected in serum-free conditioned medium from TGF-β1 treated cells (Fig. 5 d). These data suggest that in addition to transcriptional downregulation, full-length VEGFR2 is released from TGF-β1 treated endothelial cells as a mechanism to regulate angiogenesis.

## Discussion

TGF-β1 induced a dose dependent inhibition of endothelial cord formation and modulation of endothelial angiogenic receptor expression in an ALK5 dependent fashion. We observed enhanced endoglin expression, and reduced Notch1 and VEGFR2 expression, which collectively abrogated tip cell formation and stalk elongation. During sprouting angiogenesis, combined increased VEGFR2 and reduced Notch1 signaling lead to gain tip cell phenotype, and increased Notch1 signaling promotes loss of tip cell phenotype [21, 22]. Our results suggest that TGF-β1 interferes with tip/stalk cell identity when ALK5/Smad2 signaling pathways are activated.

TGF-β-mediated ALK1 signaling serves to enhance sprouting angiogenesis in part by indirectly inhibiting TGF-β/ALK5 signaling [6]. Endoglin modulates the balance between ALK1 and ALK5 signaling, favouring a pro-angiogenic phenotype [23]. However, in our study, endoglin expression was highest in cells treated with ‘anti-angiogenic’ levels of TGF-β1 (5 ng/ml and higher), which were also associated with ALK5/Smad2 activation. Interestingly, activated (phosphorylated) Smad1/5 was readily detected independent of TGF-β1 treatment. Thus, in our system, tip cell identity may be the default phenotype, which is repressed upon activation of ALK5/Smad2 signaling, possibly via loss of Notch 1 and VEGFR2 expression.

We saw concomitant loss of cell-associated full-length VEGFR2 and increased levels of CM-associated full-length VEGFR2 upon TGF-β1 exposure. Endothelial cells are known to produce an alternatively spliced VEGFR2 mRNA, leading to transcripts lacking the transmembrane domain coded for by exon 13 [24, 25]. This transcript codes for a soluble form of the receptor (sVEGFR2), which could potentially account for the presence of CM-associated VEGFR2 in our samples. However, while both full length and alternatively spliced VEGFR2 transcripts were detectable in BAEC, the full length VEGFR2 mRNA was by far the predominant isoform, and we found no evidence for a preferential shift in production of message for sVEGFR2 in TGF-β1 treated cells. Further, the CM-associated VEGFR2 was full-length protein as assessed by estimated molecular mass. Thus, the source of VEGFR2 found in conditioned medium is unlikely to be due to preferential translation of a sVEGFR2 variant mRNA upon exposure to TGF-β1.

Alternatively, VEGFR2 could be cleaved from the BAEC cell surface through proteolytic activity. The matrix metalloproteinase MMP-14 (membrane type MMP; MT1-MMP) is known to cleave the TGF-β type III receptor endoglin from endothelial cells, generating a soluble form [26]. In our study, levels of MMP-14 were weakly detectable by western blotting, and qPCR revealed C*q* values > 35 (data not shown). This, combined with the lack of detectable soluble endoglin in BAEC conditioned medium, suggests that MMP-14 cleavage of surface protein is not a significant determinant of CM-associated VEGFR2 in our system. A disintegrin and metalloprotease (ADAM) family of enzymes act as ‘sheddases’, cleaving surface bound proteins to general soluble isoforms. ADAM-17 is known to shed VEGFR2 from endothelial [27] and non-endothelial [28] cell surfaces, and VEGFR2 is a target of ADAM-10 mediated cleavage in endothelial cells [29]. Both ADAM-10 and ADAM-17 were produced by BAECs, and there was weak induction of ADAM-10 by TGF-β1 in our cells. However, such sheddase activity would generate CM-associated VEGFR2 molecules of ~130 kDa [27], but we only detected full length (~250 kDa) VEGFR2 in conditioned medium, consistent with an alternative mechanism for release of this receptor from endothelial cells. Recent reports suggest that prolonged surface residence of VEGFR2 in HUVECs leads to protease-mediated cleavage and the generation of a soluble fragment of ~100 kDa and a residual cell associated 130 kDa fragment [30]. As well, enhanced ubiquitination of VEGFR2 upon internalization in HUVECs leads to endosome-lysosome pathway mediated fragmentation into 160 and 120 kDa fragments [31, 32]. The absence of such VEGFR2 fragments in our samples suggests that TGF-β1 is not modulating VEGFR2 levels via these mechanisms in these BAECs.

There is growing evidence that VEGFR2 signals cells in an ‘autocrine’ or ‘intracrine’ fashion, leading to increased receptor activity within the cytosolic (endosome) and/or nuclear compartments [33–36]. Endothelial cells can also release exosomes containing sequestered molecules that then modulate neighbouring cells [37–39]. Endothelial cells expressing high levels of endoglin also upregulate autophagy [40]. Autophagy may enhance extracellular vesicle/exosome release via the mutivesicular body pathway, especially in serum starved endothelial cells [41, 42]. These pathways may also be relevant for tip/stalk cell specification during sprouting angiogenesis, as tips cells display enhanced VEGFR2 turnover via endocytosis and transfer from early endosomes to multivesicular bodies [42, 43]. Further, Dll4 containing exosomes can be shed from endothelial cells, and Dll4 containing exosomes modulate tip cell phenotype during sprouting angiogenesis [39, 44].

In agreement with the aforementioned findings, our results extend this activity to include the possibility that shedding of VEGFR2 containing extracellular vesicles (possibly exosomes) by vascular endothelium may be enhanced upon exposure to TGF-β1. We detected full length VEGFR2 in serum free conditioned medium along with the exosome marker HSP90. Retinal pigment epithelial cells also shed exosomes containing VEGFR2 which subsequently modulate endothelial cord formation in vitro [45], but to our knowledge this is the first report that VEGFR2 might be shed from endothelial cells themselves.

## Conclusions

Here we report that TGF-β1 alters levels of key angiogenic receptors, and interferes with tip/stalk cell identity when ALK5/Smad2 signaling pathways are activated. Our results suggest that downregulation of surface VEGFR2 and concomitant increase in VEGFR2 levels in endothelial cell conditioned medium may be a direct response to ALK5-mediated TGF-β signaling. Exosome shedding of VEGFR2 may rapidly limit the effects of angiogenic stimuli on the cells and stop further sprout formation, but the role of these events in physiological and pathological angiogenesis requires further investigation.

## Additional files

Additional file 1: Figure S1. Time course of restoration of VEGFR2 expression. A) Western blot of BAEC treated with serum free media containing 5 ng/ml TGF-β1 for 24 h, followed by recovery in serum free medium lacking TGF-β1. Samples were collected after 0, 24, 48 and 72 h recovery. Control culture (C) was treated with serum free medium lacking TGF-β1 for 24 h. B) Densitometry of western blots showing significant recovery of VEGFR2 expression by 48 h post TGF-β1 treatment. * *p* < 0.001; *N* = 2. (PDF 167 kb)
Additional file 2: Figure S2. ALK5 inhibitor SD-208 prevents TGF-β1 induced downregulation of VEGFR2 expression. Western blots showing impact of increases doses of SD-208 on BAEC treated with DMSO vehicle (0) or 5 ng/ml TGF-β1 for 24 h. (PDF 176 kb)
