# Supplementary material for: Release of endothelial cell associated VEGFR2 during TGF-β modulated angiogenesis in vitro
Source: BMC Cell Biol. 2017 Jan 23;18:10. doi: 10.1186/s12860-017-0127-y (PMC5260130; doi:10.1186/s12860-017-0127-y)
Supplement: Additional file 1: Figure S1. — Time course of restoration of VEGFR2 expression. A) Western blot of BAEC treated with serum free media containing 5 ng/ml TGF-β1 for 24 h, followed by recovery in serum free medium lacking TGF-β1. Samples were collected after 0, 24, 48 and 72 h recovery. Control culture (C) was treated with serum free medium lacking TGF-β1 for 24 h. B) Densitometry of western blots showing significant recovery of VEGFR2 expression by 48 h post TGF-β1 treatment. * p < 0.001; N = 2. (PDF 167 kb) [file 12860_2017_127_MOESM1_ESM.pdf]

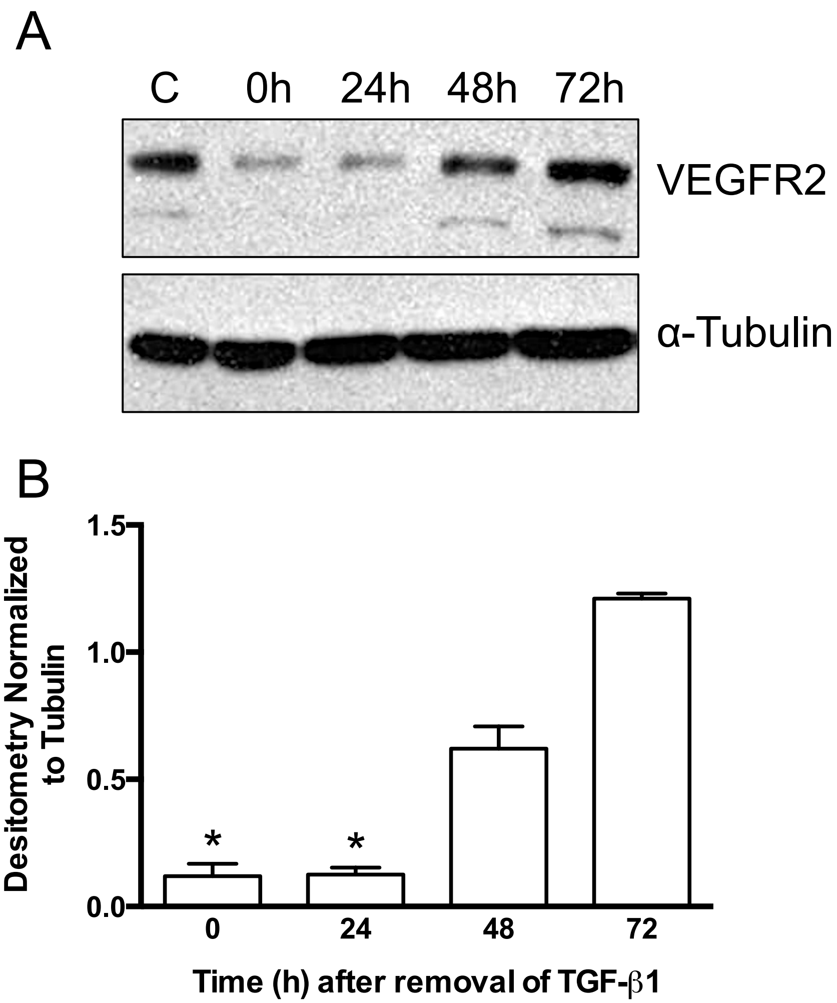

Supplemental Figure 1: Time course of restoration of VEGFR2 expression.
A) Western blot of BAEC treated with serum free media containing 5 ng/ml TGF-β1 for 24 hours, followed by recovery in serum free medium lacking TGF-β1. Samples were collected after 0, 24, 48 and 72 hours recovery. Control culture (C) was treated with serum free medium lacking TGF-β1 for 24 hours. **B**) Densitometry of western blots showing significant recovery of VEGFR2 expression by 48 hours post TGF-β1 treatment. * $p < 0.001$; $N = 2$.
