# Supplementary material for: Release of endothelial cell associated VEGFR2 during TGF-β modulated angiogenesis in vitro
Source: BMC Cell Biol. 2017 Jan 23;18:10. doi: 10.1186/s12860-017-0127-y (PMC5260130; doi:10.1186/s12860-017-0127-y)
Supplement: Additional file 2: Figure S2. — ALK5 inhibitor SD-208 prevents TGF-β1 induced downregulation of VEGFR2 expression. Western blots showing impact of increases doses of SD-208 on BAEC treated with DMSO vehicle (0) or 5 ng/ml TGF-β1 for 24 h. (PDF 176 kb) [file 12860_2017_127_MOESM2_ESM.pdf]

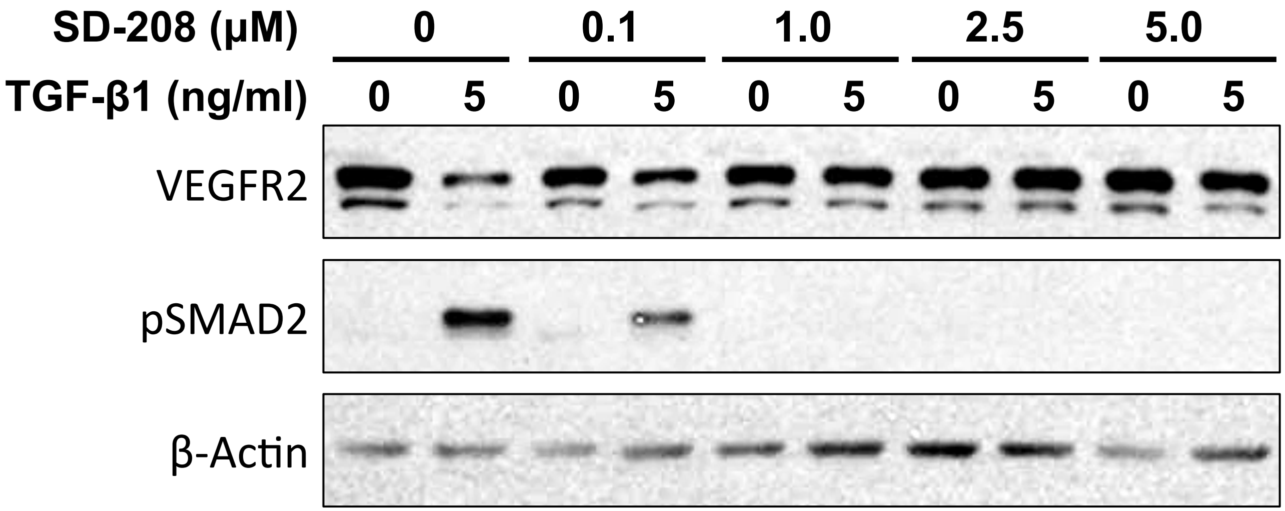

Supplemental Figure 2: ALK5 inhibitor SD-208 prevents TGF-β1 induced downregulation of VEGFR2 expression.
Western blots showing impact of increases doses of SD-208 on BAEC treated with DMSO vehicle (0) or 5 ng/ml TGF-β1 for 24 hours.
